# Value of CRP, PCT, and NLR in Prediction of Severity and Prognosis of Patients With Bloodstream Infections and Sepsis

**DOI:** 10.3389/fsurg.2022.857218

**Published:** 2022-03-07

**Authors:** Peipei Liang, Feng Yu

**Affiliations:** Department of Emergency Intensive Care Unit, The First Affiliated Hospital of AnHui Medical University, Hefei, China

**Keywords:** C-reactive protein, procalcitonin, neutrophil to lymphocyte ratio, bloodstream infection and sepsis, prognosis, condition

## Abstract

**Objective:**

To investigate the value of C-reactive protein (CRP), procalcitonin (PCT), and neutrophil to lymphocyte ratio (NLR) in assessing the severity of disease in patients with bloodstream infection and sepsis, and to analyze the relationship between the levels of three inflammatory factors and the prognosis of patients.

**Methods:**

The clinical data of 146 patients with bloodstream infection and sepsis admitted to our intensive care unit (ICU) from October 2016 to May 2020 were retrospectively analyzed. The differences in the levels of inflammatory indicators such as CRP, PCT, and NLR within 24 h in patients with bloodstream infection sepsis with different conditions (critical group, non-critical group) and the correlation between these factors and the condition (acute physiology and chronic health evaluation II, APACHE II score) were analyzed. In addition, the prognosis of all patients within 28 days was counted, and the patients were divided into death and survival groups according to their mortality, and the risk factors affecting their death were analyzed by logistic regression, and the receiver operating characteristic (ROC) curve was used to analyze the value of the relevant indicators in assessing the prognosis of patients.

**Results:**

The levels of NLR, CRP, PCT, total bilirubin (TBIL), glutamic oxaloacetic transaminase (AST), and serum creatinine (Scr) were significantly higher in the critically ill group than in the non-critically ill group, where correlation analysis revealed a positive correlation between CRP, PCT, and NLR and APACHE II scores (*P* < 0.05). Univariate logistic regression analysis revealed that CRP, PCT, NLR, and APACHE II scores were associated with patient prognosis (*P* < 0.05). Multi-factor logistic regression analysis found that PCT, NLR, and APACHE II scores were independent risk factors for patient mortality within 28 days (*P* < 0.05). ROC curve analysis found that PCT and NLR both had an AUC area > 0.7 in predicting patient death within 28 days (*P* < 0.05).

**Conclusion:**

Inflammatory factors such as NLR, CRP, and PCT have important clinical applications in the assessment of the extent of disease and prognosis of patients with bloodstream infection and sepsis.

## Background

Bloodstream infection is a serious infectious disease that occurs when pathogenic microorganisms such as bacteria and fungi invade the blood circulation through a damaged skin mucosal barrier, causing systemic disseminated infection, and septicemia and bacteremia are often referred to clinically as bloodstream infection (1, 2). Clinical practice shows (3) that patients with bloodstream infections caused by bacterial infections have a very high probability of developing sepsis, and some studies (4) suggest that patients with bacterial bloodstream infections develop rapidly and can develop septic shock within a short period of time, which can lead to multi-organ failure or even death in severe cases. The incidence of sepsis is increasing every year and has a very high mortality rate (5, 6). It has been shown (7, 8) that if patients can be treated correctly within 1 h of onset, their survival rate can be 80% or more, while if patients are treated more than 6 h after onset their probability of survival is more than 30%. Thus, early risk stratification and timely identification of critical conditions are key to improving the short-term prognosis of patients with bloodstream infection sepsis.

Currently, blood cultures are the gold standard for the diagnosis of bloodstream infections, but they are time-consuming, taking 3–7 days or more, with poor timeliness and the possibility of contaminated specimens (9). In recent years, some clinical studies have found that the levels of inflammatory factors such as calcitoninogen (PCT) and C-reactive protein (CRP) have high clinical value in the early diagnosis of sepsis and determination of the disease (10, 11). PCT and CRP are two important inflammatory cytokines that are involved in apoptosis and can enable cell lysis, and their levels are significantly increased when the organism is infected with bacteria, but the relevance of changes in their levels has been less studied in patients with sepsis due to bloodstream infection (12). In addition, studies (13, 14) have shown that the neutrophil/lymphocyte ratio (NLR) can be used to predict sepsis bloodstream infections and help distinguish between different pathogenic species, but it is not widely accepted by clinicians as a definitive marker of infection and the exact threshold value is controversial. Therefore, this study was conducted to investigate the correlation between PCT, CRP, and NLR and the prognostic value of bloodstream infection in patients with sepsis, in order to provide a basis for early screening of high-risk patients and to guide clinical treatment.

## Information and Methods

### Inclusion Criteria

One hundred and forty-six patients with bloodstream infection sepsis (positive blood cultures) admitted to our intensive care unit (ICU) from October 2016 to May 2020 were selected, and only clinical data from the first admission were selected for repeat ICU admissions during the study period. Inclusion criteria: (1) referring to the International Consensus on the Definition of Sepsis and Infectious Shock, Third Edition (Sepsis-3) published in 2016 (15), the criteria for sepsis diagnosis were (i) identification of suspected infection; (ii) presence of a sequential organ failure score (SOFA) change ≥ 2 points; (2) the patient's age was ≥18 years; (3) the patient's case data and biochemical findings after admission were complete and free of defects; (4) two or more positive blood cultures, or two sites with the same positive blood culture and the same causative organism; (5) the patient's serum inflammatory factors PCT, CRP and NLR levels were measured within 24 h after admission. Exclusion criteria: (1) patients who had been hospitalized for <24 h; (2) patients with other viral or bacterial infections; (3) patients with immune deficiency, malignancy, hematologic disorders, or severe psychiatric disorders; (4) patients who had been treated with antibiotics prior to initial admission; (5) women who were breastfeeding or pregnant; (6) patients or family members who had requested that treatment be abandoned.

### General Information

The clinical data of 146 patients with sepsis from bloodstream infection, 60 women and 86 men, aged 18–94 (62.45 ± 14.16) years, included in the study were retrospectively analyzed. Information on gender, age, site of infection, comorbidities [multiple organ failure syndrome (MODS), acute respiratory distress syndrome (ARDS)], underlying diseases (chronic obstructive pulmonary disease, hypertension, diabetes mellitus), blood count [leucocytes (WBC), total bilirubin (TBIL), D-Dimer (D-D), Alanine transaminase (ALT), aspartate transaminase (AST), B-type natriuretic peptide (BNP), platelet count (PLT), serum creatinine (Scr)], inflammatory factor levels (CRP, PCT, and NLR), type of infectious strain, and APACHE II score at 24 h of admission were collected from all patients by case collection.

### Grouping Methods

The Acute Physiology and Chronic Health Rating Scale (APACHE II) assessment was completed within 24 h after patients were admitted to the ICU, and patients were divided into a critical group (67 patients with an APACHE II score of 20 or more) and a non-critical group (79 patients with an APACHE II score of <20) based on their scores. Patients were observed starting from the time of admission to the hospital and discharged with improvement or death within 28 days as the end point. The prognosis of all patients was counted and divided into a death group (56 patients) and a survival group (90 patients) according to their prognosis. Among them, survival means patients who improved after treatment in ICU and were transferred to other departments for further treatment or discharged from the hospital; death includes patients who died after ineffective treatment and those who were automatically discharged.

### Statistical Processing

All data collected were statistically analyzed and plotted using SPSS 22.0 and Prism 8.0. The *t*-test, expressed as mean ± standard deviation (x ± s), was used for measures that conformed to normal distribution and homogeneity of variance. For the count data, the chi-square test or Fisher's exact test was performed. Pearson model was used to analyze the correlation between each index and the condition. Independent risk factors affecting prognosis were analyzed by one-way and multi-way logistic regression, and the ROC curve was used to evaluate the diagnostic sensitivity, specificity, and optimal cutoff value of each index. *p* < 0.05 indicated that the difference was statistically significant.

## Results

### Comparison of General Data and Laboratory Indicators of Patients With Different Conditions

We compared the general data and laboratory indices of patients with sepsis from severe bloodstream infection with those of patients with sepsis from non-severe bloodstream infection. The results were no statistically significant differences between the two groups in terms of gender, age, pulmonary infection, abdominal infection, concurrent MODS, concurrent ARDS, underlying disease, strain of infection, WBC level, platelet count, ALT, D-D, BNP, and BUN levels (*P* > 0.05). The levels of NLR, CRP, PCT, TBIL, AST, and Scr were higher in patients with severe bloodstream infection and sepsis than in the non-critical group, and the differences were statistically significant (*P* < 0.05; Table 1).

**Table 1:** Comparison of general data and laboratory indicators of patients with different conditions [(x ± s), *n* (%)].

**Information**		**Critical group (*n* = 67)**	**Non-critical group (*n* = 79)**	**χ^2^/t value**	***P-*value**
Gender (male)		38 (56.72)	48 (60.76)	0.245	0.621
Age (years)		63.32 ± 13.45	61.57 ± 15.16	0.903	0.369
Infection status	Pulmonary infection	47 (70.15)	62 (78.48)	2.094	0.148
	Abdominal infection	10 (14.93)	16 (20.25)	0.703	0.402
Complicated MODS		10 (14.93)	13 (16.46)	0.064	0.800
Complicated ARDS		7 (10.45)	13 (46.46)	1.107	0.293
Underlying disease	COPD	4 (5.97)	8 (10.13)	0.830	0.362
	Hypertension	22 (32.84)	36 (45.57)	2.455	0.117
	Diabetes mellitus	8 (11.94)	12 (15.19)	0.324	0.569
Types of bacteria	Gram negative bacteria	32 (47.76)	45 (56.96)	1.231	0.267
	Gram positive bacteria	28 (41.79)	41 (51.90)	1.486	0.222
NLR		27.32 ± 16.33	12.58 ± 7.14	7.250	0.000
CRP (mg/L)		143.56 ± 56.28	89.73 ± 32.51	7.204	0.000
PCT (ng/mL)		4.79 ± 1.73	1.58 ± 0.80	14.744	0.000
WBC (×10^9^/L)		15.57 ± 8.24	13.26 ± 10.33	1.475	0.142
PLT (×10^9^/L)		130.26 ± 62.24	113.51 ± 52.33	1.767	0.079
TBIL (μmol/L)		34.65 ± 42.13	21.83 ± 29.54	2.152	0.033
AST (U/L)		84.23 ± 33.16	56.77 ± 27.56	5.465	0.000
ALT (U/L)		37.26 ± 13.24	35.58 ± 11.48	0.821	0.413
D-D (mg/L)		4.24 ± 3.10	4.05 ± 3.25	0.360	0.720
BNP (ng/L)		3246.47 ± 3717.52	2535.24 ± 3017.28	1.276	0.204
BUN (mmol/L)		12.59 ± 5.78	11.34 ± 6.04	1.271	0.206
Scr (μmol/L)		208.13 ± 114.58	164.32 ± 132.26	2.119	0.036

### Analysis of the Correlation Between Laboratory Indicators and the Degree of Sepsis in Bloodstream Infections

**Figure 1:**
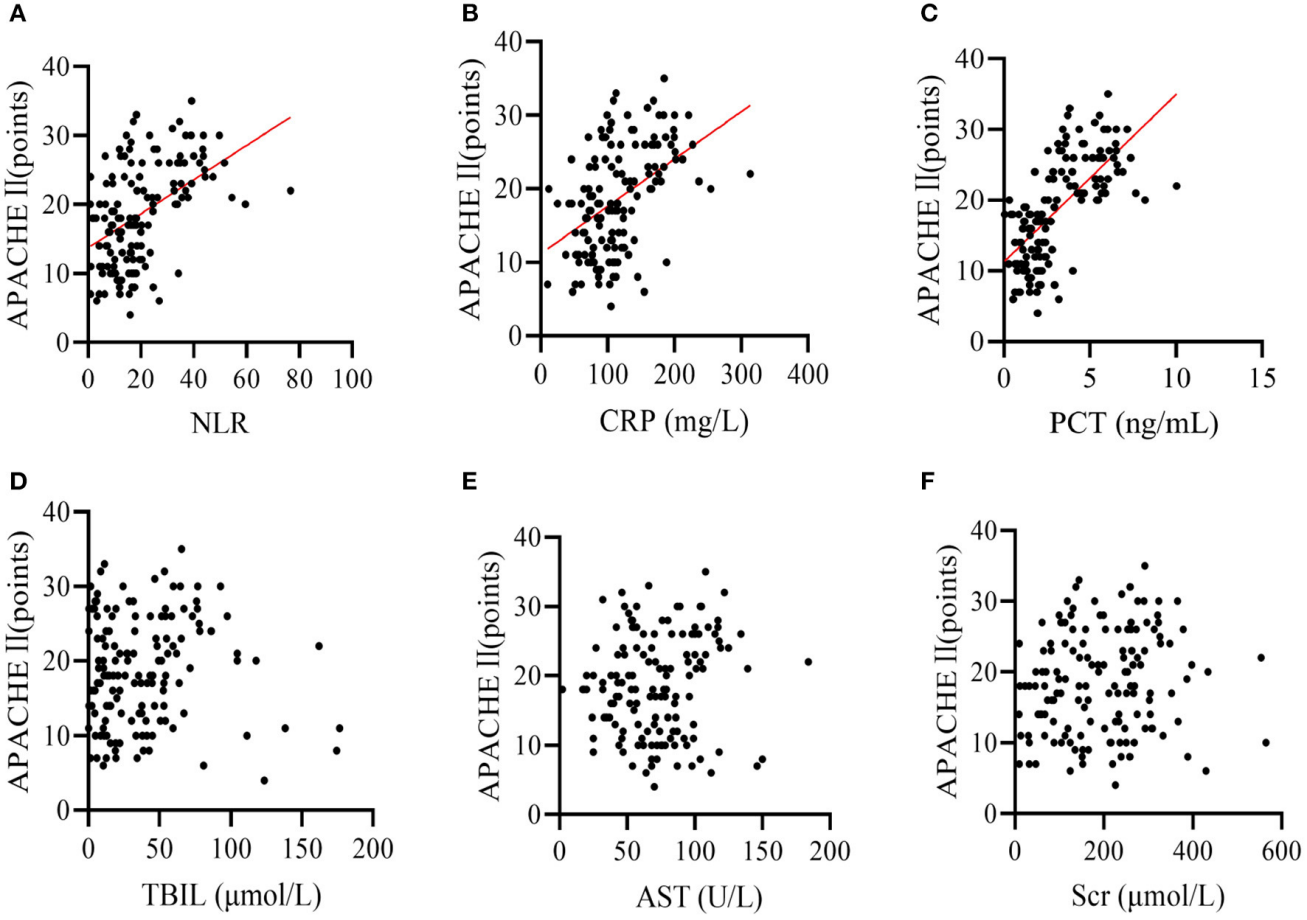
Pearson correlation analysis showed that the levels of **(A)** NLR, **(B)** CRP, and **(C)** PCT were positively correlated with the severity of disease (APACHE II) in patients with bloodstream infection sepsis (*r* values of 0.468, 0.456, and 0.670 respectively; all *P* values < 0.001); **(D)** TBIL, **(E)** AST, and **(F)** SCR levels were not linearly correlated with the severity of disease in patients with bloodstream infection sepsis (*r* values of 0.017, 0.101, and 0.117 respectively, *P* values of 0.838, 0.223, and 0.159 respectively).

Pearson correlation analysis showed that NLR, CRP and PCT levels were positively correlated with severity of disease in patients with bloodstream infection sepsis (*r*-values were 0.468, 0.456, and 0.670, respectively; all *P* < 0.001), while TBIL, AST, and Scr levels were not linearly correlated with severity of disease in patients with bloodstream infection sepsis (*r*-values were 0.017, 0.101, and 0.117, *p*-values were 0.838, 0.223, and 0.159, respectively; Figures 1A–F).

### Comparison of General Data and Laboratory Indicators of Patients With Different Prognosis

We compared the general data and laboratory indices of patients in the death group with those in the survival group. The results were no statistically significant differences between the two groups in terms of gender, age, abdominal infection, underlying disease, strain of infection, WBC level, platelet count, ALT, D-D, BNP, BUN, Scr, and TBIL levels (*P* > 0.05). The incidence of pulmonary infections, MODS and ARDS, the levels of NLR, CRP, PCT, and AST, and the APACHE II score were significantly higher in the death group than in the survival group (*P* < 0.05; Table 2).

**Table 2:** Comparison of general data and laboratory indicators of patients with different prognosis [(x ± s), *n* (%)].

**Information**		**Death group (*n* = 56)**	**Survival group (*n* = 90)**	**χ^2^/t value**	***P-*value**
Gender (male)		36 (64.29)	50 (55.56)	0.016	0.900
Age (years)		60.23 ± 17.46	55.72 ± 17.65	1.507	0.134
Infection status	Pulmonary infection	48 (85.71)	61 (67.78)	5.870	0.015
	Abdominal infection	8 (14.29)	18 (20.00)	0.770	0.380
Complicated MODS		14 (25.00)	9 (10.00)	5.852	0.016
Complicated ARDS		12 (21.43)	8 (8.89)	4.592	0.032
Underlying disease	COPD	6 (10.71)	6 (6.67)	1.124	0.289
	Hypertension	19 (33.93)	39 (43.33)	1.275	0.259
	Diabetes mellitus	6 (10.71)	14 (15.56)	0.684	0.408
Types of bacteria	Gram negative bacteria	33 (58.93)	44 (48.89)	2.576	0.109
	Gram positive bacteria	23 (41.07)	46 (51.11)	1.396	0.237
NLR		29.37 ± 12.24	13.52 ± 8.22	9.360	0.000
CRP (mg/L)		150.59 ± 57.37	106.24 ± 62.25	4.312	0.000
PCT (ng/mL)		9.43 ± 4.25	2.56 ± 1.61	13.844	0.000
WBC (×10^9^/L)		15.02 ± 11.34	12.57 ± 9.43	1.411	0.160
PLT (×10^9^/L)		132.88 ± 58.42	118.25 ± 48.37	1.639	0.103
TBIL (μmol/L)		34.77 ± 41.82	23.98 ± 31.25	1.779	0.078
AST (U/L)		82.56 ± 31.43	58.49 ± 30.24	4.607	0.000
ALT (U/L)		36.11 ± 12.25	36.59 ± 13.12	0.220	0.826
D-D (mg/L)		4.92 ± 3.21	3.89 ± 3.41	1.815	0.072
BNP (ng/L)		3,310.25 ± 3,822.01	2,843.53 ± 3,452.24	0.762	0.447
BUN (mmol/L)		13.34 ± 6.82	12.25 ± 7.27	0.902	0.369
Scr (μmol/L)		196.34 ± 119.24	173.25 ± 124.58	1.107	0.270
APACHE II (points)		25.24 ± 5.15	20.87 ± 4.23	5.578	0.000

### Logistic Regression Analysis of Risk Factors Affecting 28 Days Mortality in Patients With Bloodstream Infection and Sepsis

Patients' prognosis at 28 days was used as the dependent variable (survival = 0, death = 1) and relevant influencing factors were used as independent variables (see Table 3 for assignments) to enter a single multifactorial analysis. Logisti univariate analysis showed that NLR level, CRP level, PCT, and APACHE II score were associated with 28 days mortality in patients with bloodstream infection and sepsis (*P* < 0.05). Further multiple regression analysis of the indicators that differed in the univariate analysis showed that NLR level, PCT level and APACHE II score were independent risk factors for death at 28 days in patients with bloodstream infection and sepsis (*P* < 0.05; Tables 3, 4).

**Table 3:** Assignment table.

**Indicator**	**Assignment**
Pulmonary infection	Yes = 0, No = 1
Complicated MODS	Yes = 0, No = 1
Complicated ARDS	Yes = 0, No = 1
NLR	Continuous variables
CRP	Continuous variables
PCT	Continuous variables
AST	Continuous variables
APACHEII	Continuous variables

**Table 4:** Logistic regression analysis of risk factors affecting 28 days mortality in patients with bloodstream infection and sepsis.

**Indicators**	**Single-factor**			**Multi-factor**		
	**OR**	**95% CI**	***P*-value**	**OR**	**95% CI**	***P*-value**
Pulmonary infection	1.379	0.799~2.377	0.623	–	–	–
Complicated MODS	1.525	0.695~3.347	0.512	–	–	–
Complicated ARDS	1.511	0.746~3.049	0.324	–	–	–
NLR	1.923	1.575~2.349	0.012	1.669	1.402~1.987	0.009
CRP	1.958	1.030~3.724	0.046	1.060	0.957~1.173	0.206
PCT	1.684	1.347~2.105	0.038	1.687	1.312~2.151	0.031
AST	1.132	0.934~1.372	0.344	–	–	–
APACHE II	2.387	1.223~4.657	0.006	2.270	1.237~4.169	0.007

### Analysis of the Predictive Value of Relevant Indicators on the Prognosis of Patients With Bloodstream Infection and Sepsis

**Figure 2:**
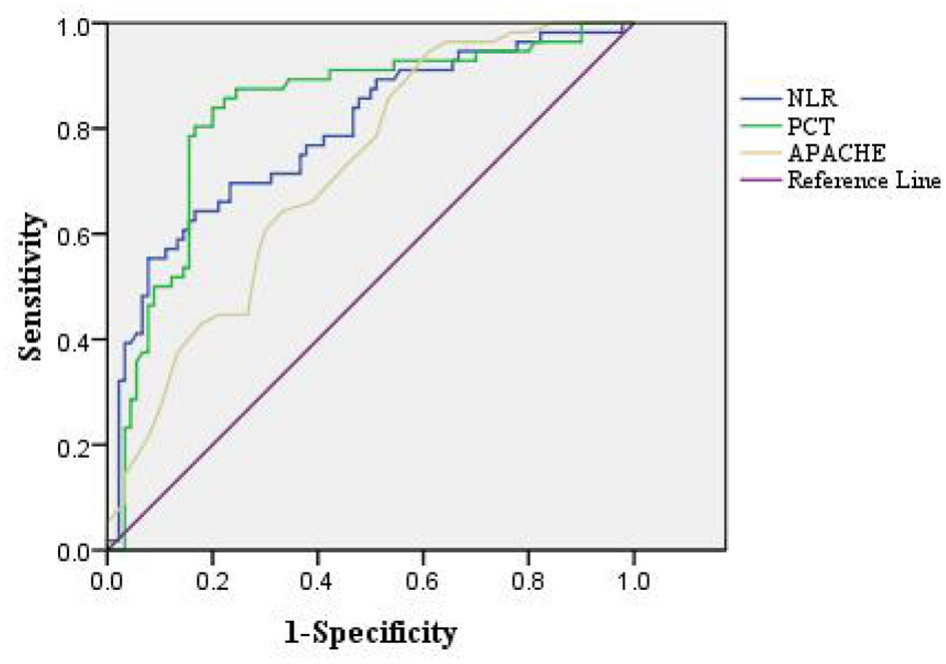
ROC curve of the predictive value of relevant indicators for 28 days death in patients with bloodstream infection and sepsis.

The area under the curve (AUC) for NLR, PCT, and APACHE II scores to predict 28 days death in patients with bloodstream infection and sepsis were 0.791 (95% CI: 0.714–0.868), 0.830 (95% CI: 0.758–0.902), and 0.718 (95% CI: 0.636–0.800), respectively, all with *P* < 0.05 (Table 5; Figure 2).

**Table 5:** Analysis of the predictive value of relevant indicators on the prognosis of patients with bloodstream infection and sepsis.

**Indicators**	**AUC**	**95% CI**	**Cut-off**	**Sensitivity (%)**	**Specificity (%)**
NLR	0.791	0.714~0.868	0.476	64.30	83.30
PCT	0.830	0.758~0.902	0.639	83.90	80.00
APACHE II	0.718	0.636~0.800	0.326	89.30	43.30

## Discussion

Bloodstream infection is usually defined as the same patient's blood samples obtained from different parts of the body to culture the same bacteria, and the patient shows symptoms and signs related to the pathogenic bacteria, it is a serious infectious disease that can spread throughout the body and is often life-threatening (16). Sepsis is usually triggered by dysregulation of the organism's response to infection and is an acute organ dysfunction resulting from an abnormal immune response of the host to infection (17). Some clinical studies (18) have shown that among the various types of infections that cause sepsis, bloodstream infection is the most important factor in the death of patients with sepsis. And with the gradual increase in the incidence of sepsis after bloodstream infection in China in recent years, the research on the condition, prognosis assessment and prevention and treatment of sepsis caused by bloodstream infection has become a hot spot of concern for clinical workers.

Most scholars (19, 20) currently believe that the development, progression and prognosis of sepsis in bloodstream infections are related to the inflammatory response and the immune function status of the body. It has been reported (21) that serum levels of PCT and CRP, two important inflammatory cytokines involved in apoptosis and capable of causing cell lysis, are significantly increased when the body is infected with bacteria. PCT, a precursor of calcitonin, is a 116-amino acid glycoprotein that can be detected as a significant increase in PCT levels in the presence of bacterial infections, but is rarely seen in viral infections and is a sensitive substance for identifying bacterial and viral infections (22). CRP is an acute phase reaction protein, mainly synthesized and released by the liver, which slowly increases to hundreds or thousands of times its normal level after 8–12 h when the organism is invaded by pathogenic microorganisms or stimulated by inflammation, and gradually returns to normal level after reasonable treatment, which can reflect the severity of infection to some extent (23). As for the relationship between NLR and sepsis, some studies (24) have suggested that leukocytosis and lymphopenia are a distinctive feature of severe sepsis, and some foreign scholars (25, 26) have suggested that NLR can be used as a marker for the early detection and diagnosis of sepsis and that NLR is more effective than conventional inflammatory biomarkers. This study focused on analyzing the differences in the levels of PCT, CRP, and NLR in patients with bloodstream infection sepsis and correlating them with the concurrently rated APACHEII to provide a reference basis for early clinical assessment of the severity of sepsis patients and their prognosis.

By evaluating the value of PCT, CRP, and NLR in assessing the condition of patients with sepsis due to bloodstream infection, it was found that the levels of NLR, CRP, and PCT were significantly higher in patients with sepsis due to severe bloodstream infection than in the non-severe group. APACHE II is currently the most commonly used disease severity scoring system in ICU, and to some extent the magnitude of its score can indicate the prognosis of the disease, with the higher the score the worse the prognosis (27). Therefore, this study further correlated the inflammatory factors PCT, CRP, and NLR with APACHE II scores and found that there was a positive correlation between serum PCT, CRP, and NLR and APACHE II scores. This suggests that the severity of the disease in patients with bloodstream infection sepsis can be effectively reflected by the inflammatory factors PCT, CRP, and NLR. Meanwhile, we compared patients with different prognosis of bloodstream infection sepsis and found that higher levels of PCT and NLR tended to suggest higher sepsis mortality, and both had comparable predictive value for 28 days death in patients with bloodstream infection and sepsis.

In conclusion, NLR, CRP, and PCT can effectively reflect the severity of patients with bloodstream infection sepsis, and higher levels of NLR, CRP, and PCT are associated with severe disease. In addition NLR, PCT, and APACHE II scores are independent risk factors for predicting death in 28 days of bloodstream infection sepsis, and early monitoring of inflammatory factors for patients with bloodstream infection sepsis can be used as a marker for assessing the extent of patients' disease as well as their prognosis.

## Data Availability Statement

The original contributions presented in the study are included in the article/supplementary material, further inquiries can be directed to the corresponding author/s.

## Ethics Statement

The studies involving human participants were reviewed and approved by the Ethics Committee of The First Affiliated Hospital of AnHui Medical University. The patients/participants provided their written informed consent to participate in this study.

## Author Contributions

FY was the supervisor of this study. All authors of this study made equal contributions, including the design of the study, the conduct of the experiments, and the writing of the paper.

## Conflict of Interest

The authors declare that the research was conducted in the absence of any commercial or financial relationships that could be construed as a potential conflict of interest.

## Publisher's Note

All claims expressed in this article are solely those of the authors and do not necessarily represent those of their affiliated organizations, or those of the publisher, the editors and the reviewers. Any product that may be evaluated in this article, or claim that may be made by its manufacturer, is not guaranteed or endorsed by the publisher.
